# Assessment of autoantibodies in paediatric population with primary immunodeficiencies: a pilot study

**DOI:** 10.1186/s12865-023-00543-6

**Published:** 2023-06-03

**Authors:** Karolina Pieniawska-Śmiech, Aleksandra Lewandowicz-Uszyńska, Magdalena Zemelka-Wiacek, Marek Jutel

**Affiliations:** 1 Department of Clinical Immunology, Wroclaw Medical University, 50-368 Wroclaw, Poland; 2 Department of Clinical Immunology and Paediatrics, Provincial Hospital J.Gromkowski, 51-149 Wroclaw, Poland; 3 3rd Department and Clinic of Paediatrics, Immunology and Rheumatology of Developmental Age, Wroclaw Medical University, 50-367 Wroclaw, Poland; 4 ALL-MED Research Institute, 53-201 Wroclaw, Poland

**Keywords:** Autoantibody, Autoimmunity, Coeliac disease, Immune dysregulation, Inborn errors of immunity, Primary immunodeficiency

## Abstract

**Background:**

The correlation between primary immunodeficiencies (PIDs) and autoimmunity shows ethnic and geographical diversity. The aim of our study was to accumulate more data in paediatric PID population.

**Methods:**

58 children aged 1–17 and with PID (study group) and 14 age-matched immunocompetent individuals (control group) were included in the study. Serum levels of 17 different specific IgG antibodies against autoantigens were measured by means of a quantitative enzyme immunoassay. Immunoglobulin levels were analysed in relation to a detailed medical examination.

**Results:**

Autoantibodies against one or more antigens were detected in the sera of 24.14% (n = 14) subjects in the study group. The most frequent were anti-thyroid peroxidase (anti-TPO) antibodies (n = 8; 13.8%). Anti-TPO antibody levels were elevated more often in PID patients with a positive family history of autoimmune diseases (p = 0.04). The screening for anti-deamidated gliadin peptide (DGP) and anti-tissue transglutaminase (tTG) antibodies in our series allowed identifying two previously undiagnosed cases of coeliac disease in PID patients. There was no statistically significant difference between the study and the control group in terms of the autoantibodies prevalence.

**Conclusions:**

This study provides data on the prevalence of autoantibodies in paediatric population diagnosed with PID. Selected autoantibodies (i.e. anti-tTG, anti-DGP) might be useful for the screening of PID to avoid the delay of diagnosis of an autoimmune disease.

**Supplementary Information:**

The online version contains supplementary material available at 10.1186/s12865-023-00543-6.

## Background

Inborn errors of immunity (IEI) are a heterogeneous group of inherited diseases caused by monogenic germline mutations and result in the loss or gain of the function of the encoded protein. Individually, most IEI occur rarely, but collectively they are more common than is generally believed [1, 2]. The description of clinical phenotypes is constantly updated in the growing field of primary immunodeficiencies (PIDs) [1, 3–5]. The 55 novel gene defects reported in the last IEI update bring the total number of IEI to 485. In practice, IEI are associated with increased susceptibility to infectious diseases (especially severe, atypical, and/or recurrent infections). The diversity of autoimmune, autoinflammatory, allergic and/or malignant phenotypes is also associated with IEI [1].

The correlation between PIDs and autoimmunity has been extensively analysed [6–12]. In the past, PIDs and autoimmune diseases (AD) were considered independent, or even polar opposites [7, 8]. Nowadays, PIDs are regarded as unprecedented models connecting defined monogenic defects with clinical manifestations of incorrect immune regulation and a clinical picture characterised by infectious complications and autoimmunity [5, 6, 13]. The pathogenic process leading to the autoimmunity is complex and includes disturbed B cell differentiation and germ‐center reactions, altered T cell central or peripheral tolerance, uncontrolled lymphocyte proliferation and differentiation, disturbances in Treg/Th17 balance, dysfunctional complement and innate immune activation, and the defective clearance of the infectious agents [6, 11].

Scientific data suggests that autoimmunity may be associated with PIDs in a significant proportion of patients [14–18]. Autoimmune manifestations are observed frequently in patients with primary antibody deficiencies (PADs), but are also reported in individuals with combined immunodeficiency disorders (CIDs) [19]. Moreover, many PIDs are associated with defects in the frequency and function of T regulatory (Treg) cells, as well as with the production of autoantibodies (AA). The IUIS classification features an independent category named”diseases of immune dysregulation”. A regulatory disorder can cause abnormal activation and expansion of immune cells, leading to autoimmunity, hyperinflammation and malignant proliferation [8]. Those might result in a bad prognosis in patients with immune dysregulation compared to those with only high susceptibility to infections [13, 14]. Due to the coexistence of autoimmunity and immunodeficiency in some cases, the treatment may be challenging and requires a personalised approach [18].

To this date, several studies have been conducted to analyse the frequency as well as the mechanisms of an AD in PIDs [14, 15]. Most of the published studies were concentrated on clinical presentation of AD. However, their interpretation is difficult due to ethnic and geographical diversity. More data is required for conclusive evidence. The occurrence of AA in paediatric PID patients has not been well established. Considering its poor prognosis and difficult treatment, a better understanding of the wide spectrum of immune dysregulation in IEI is required for precise and timely diagnosis, as well as disease monitoring and therapy. The aim of our study was to collect more data about the occurrence of AA in paediatric PID population.

## Methods

The study was approved by the Bioethical Commission of Wrocław Medical University, Wrocław, Poland. Patients included in the study group remained under the care of the Clinical Immunology and Paediatric Department of the J. Gromkowski Provincial Hospital in Wrocław due to the previously diagnosed PIDs (by a trained immunologist, according to the IUIS classification and ICD-10). Age-matched immunocompetent individuals constituted the control group (Additional file 1: Table S1). The control group consisted of patients with occasional or mild recurrent infections (less than 8 per 12 months, often treated without antibiotics, mostly upper respiratory tract infections) diagnosed in the Department of Immunology and Paediatrics who did not show abnormalities in immunological tests and did not meet IUIS criteria for inborn errors of immunity (IEI).

Blood samples were collected between 2020 and 2021. All parents or legal guardians and patients over 16 years of age signed informed consent before participating in the research.

The control group numbered 14 subjects (n = 7 boys, n = 7 girls) whose age ranged from 1 year old up to 16 years old (median age = 7; mean age = 7). The study group included 58 subjects (n = 36 boys, n = 22 girls) aged 1–17 (median age = 7; mean age = 8). Patients with predominantly antibody deficiencies (PADs; n = 46; 79.31%) comprised the majority of the study group, followed by individuals diagnosed with CIDs associated with syndromic features (n = 5; 8.62%). The baseline characteristics of the patients are presented in Additional file 2: Table S2. During the study period, eight patients (13.79%) were undergoing immunoglobulin replacement therapy (IRT), one (1.67%) completed treatment with high doses of immunoglobulins (Ig) due to immune thrombocytopenia, while 49 (84.48%) did not receive IRT (IRT−). Individuals receiving IRT (IRT +) included patients with X‐linked agammaglobulinemia (XLA; n = 1), common variable immunodeficiency (CVID; n = 3), IgG subclass and IgM deficiency (n = 1), IgG deficiency (n = 1), as well as ataxia-telangiectasia (A-T; n = 2). Doses of Ig were individualized and were within 0.2–0.8 g/kg. In all cases, substitution therapy had started before the inclusion in the current study. The blood samples were collected immediately before Ig infusion.

During the study, detailed medical history of the patients was collected, in particular place of residence (village, town, city), diagnosis of autoimmune disease, family history of autoimmune diseases, recurrent respiratory tract infections, PID complications, presence of hepato- and/or splenomegaly and/or lymphadenopathy, medications taken, diagnosis of allergic diseases, results of tests conducted before inclusion in the IRT (if available).

The following parameters were evaluated based on venous blood samples during the study period among the study group: (1) concentration of specific IgG antibodies against antigens: Ro/SS-A 52, La/SS-B, Scl-70, PM/Scl-100, Sm, PCNA, dsDNA, ribosomal P protein, CENP-B, AMA M2, MPO, PR3, TPO, TG, deamidated gliadin peptides (DGP), tissue transglutaminase (tTG), intrinsic factor (IF); (2) haemoglobin (Hgb) level; (3) white blood cell (WBC) count; (4) platelet count; (5) IgG and IgG subclass concentration; (6) anti-tTG IgA in part of the study group. Anti-tTG IgA and anti-DGP IgA together with total IgA were measured in the control group according to the guidelines for immunocompetent subjects [38].

Autoantibodies were measured by means of a quantitative enzyme immunoassay—the Polycheck ® test (Biocheck GmbH, Munster, Germany). The threshold for a positive result was ≥ 0.80 kU/L. According to the assay characteristics, the sensitivity of an assay was 96.4% and the specificity was 97%. The IgG concentration was evaluated using immunoturbidimetry, while IgG subclass levels by the nephelometric method. Immunoglobulin levels, Hgb level, WBC count and platelet count reference ranges depended on the age of the tested individual.

A statistical analysis of data was conducted based on a spreadsheet prepared in Microsoft Office Excel (Microsoft Corp. Washington, WA, USA) and Statistica v.13—Mann–Whitney U test for non-parametric quantitative data and Pearson’s chi-squared test for qualitative data. The significance level was established at α = 0.05. A p-value less than 0.05 was considered statistically significant.

## Results

### Prevalence of autoantibodies

Autoantibodies against one or more antigens from the used kit were detected in sera of 24.14% (n = 14) PID patients (Table 1). Most of patients had positive AA against one (n = 7) or two (n = 4) antigens. The majority of individuals with positive AA were boys (n = 9), however, there was no statistically significant difference in terms of gender (p = 0.844). There was no statistically significant difference between PID patients and healthy controls in case of antinuclear antibodies (ANA; p = 0.551) and anti-thyroid peroxidase (anti-TPO; p = 0.609).

**Table 1:** Characteristics of PID patients with positive autoantibodies (> = 0.80 kU/l)

Patient no.	Age (years)	Type of PID	Reported clinical symptoms and signs	Detected autoantibodies	IgG (mg/dl)	IRT	Family history of autoimmune diseases	Diagnosis of autoimmune disease
1	17	PAD (selective IgA deficiency)	Hepatomegaly Splenomegaly	Anti-Sm = 67 kU/l Anti-SS-B = 0.92 kU/l	1386 [680–1530]	No	No	Not confirmed
2	13	PAD (selective IgA deficiency)	Chronic diarrhoea	Anti-DGP = 1.4 kU/l	1472 [518–1284]	No	Yes (coeliac disease)	CD—not confirmed with other tests
3	6	PAD (selective IgA deficiency)	Failure to thrive	Anti-TPO = 3.3 kU/l Anti-IF = 1.1	1296 [540–1822]	No	Yes (Hashimoto disease)	Not confirmed
4	6	PAD (selective IgA deficiency)	Failure to thrive	Anti-TPO = 4 kU/l Anti-TG = 0.53 kU/l	1376 [553–1631]	No	Yes (Hashimoto disease)	Not confirmed
5	8	PAD (IgM deficiency)	Chronic diarrhoea Hepatomegaly	Anti-DGP = 7.4 kU/l Anti-tTG = 1.1 kU/l Anti-tTG IgA = 140 AU/ml	823 [553–1631]	No	Yes (coeliac disease)	Confirmed CD
6	4	PAD (IgG subclass deficiency)	Chronic diarrhoea Failure to thrive Coeliac disease	Anti-DGP = 14 kU/l Anti-tTG = 1.1 kU/l Anti-tTG IgA = 1140 AU/ml	480 [468–1150]	No	No	CD (diagnosed before the study)
7	7	PAD (IgG subclass deficiency)	Hypothyroidism Coeliac disease	Anti-TPO = 10 kU/l	751 [553–1631]	No	Yes (psoriasis)	Under endocrinologist care
8	9	PAD (IgG subclass deficiency)	No	Anti-TPO = 3.4 kU/l	826 [540–1822]	No	Yes (Hashimoto disease)	Not confirmed
9	6	PAD (IgG subclass deficiency)	Failure to thrive	Anti-TPO = 1.3 kU/l	964 [553–1232]	No	No	Not confirmed
10	15	PAD (X-linked agammaglobulinemia)	No	Anti-TPO = 1.1 kU/l	872 [518–1284]	Yes	Yes	Not confirmed
11	11	Ataxia-telangiectasia	No	Anti-TPO = 0.92 kU/l	906 [553–1631]	Yes	No	Not confirmed
12	10	Lymphocyte T deficiency	Skin eczema	anti-Sm = 1.8 kU/l	939 [553–1631]	No	Unknown	Not confirmed
13	4	Phagocyte number and/or function deficiency	No	Anti-TPO = 6.2 kU/l Anti-TG = 1 kU/l	665 [468–1150]	No	Yes	Not confirmed
14	3	Other: lymphocyte T deficiency, chronic neutropenia	Papulovesicular eczema	Anti-DGP = 8.4 kU/l Anti-tTG IgA = 18.7 AU/ml	1098 [540–1822]	No	No	Confirmed CD

Anti-SS-B—anti Sjogren’s Syndrome B; CD—coeliac disease; DGP—deamidated gliadin peptides; IF—intrinsic factor; Ig—immunoglobulin; IRT—immunoglobulin replacement therapy; No—number; PAD—predominantly antibody deficiency; PID—primary immunodeficiencies; Sm—Smith antigen; TG—thyroglobulin; TPO—thyroid peroxidase; tTG—tissue transglutaminase

**Fig.1:**
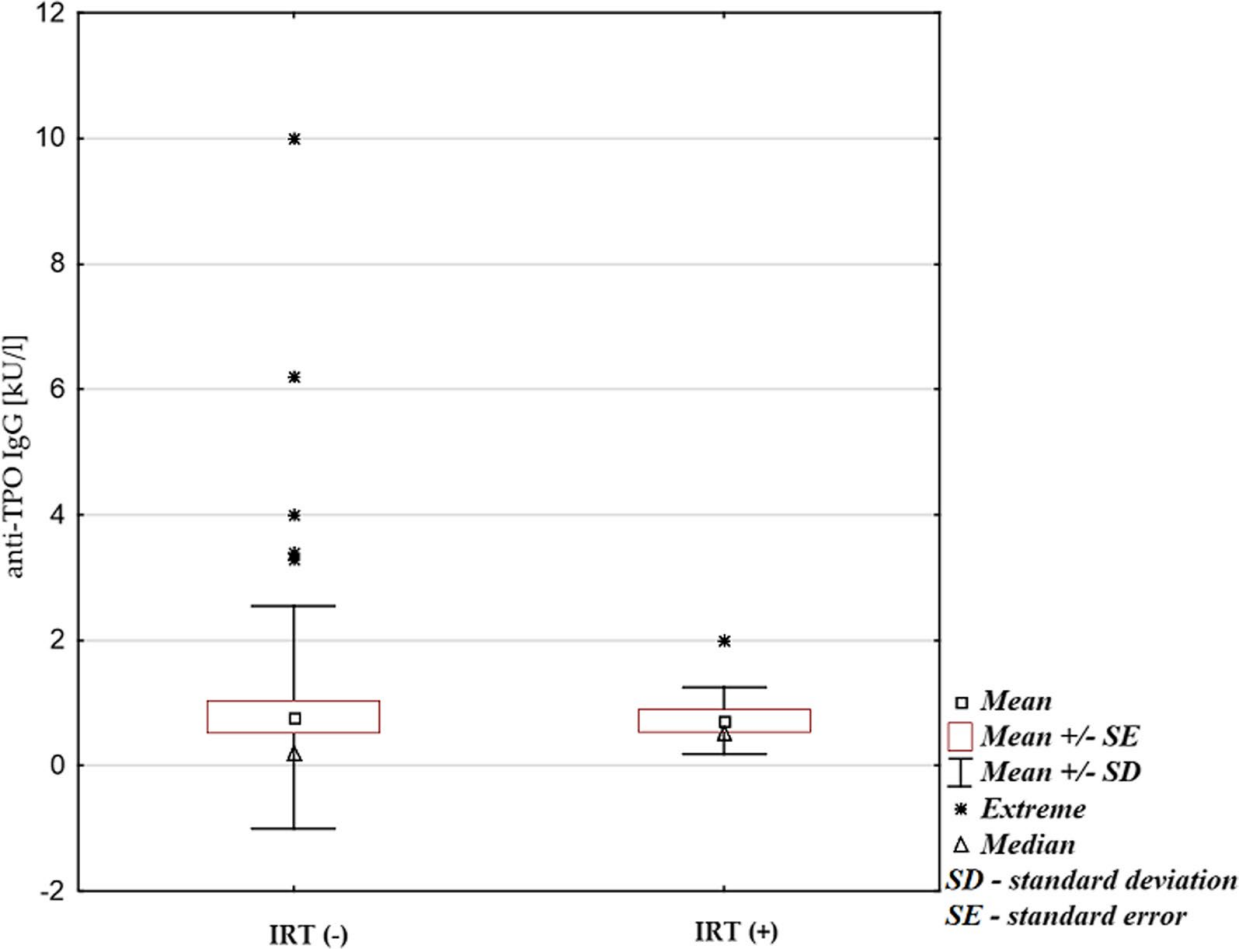
Anti-TPO antibodies level in patients undergoing (IRT +) and not undergoing (IRT −) immunoglobulin replacement therapy (IRT; p = 0.0009; Mann–Whitney U test for non-parametric quantitative data)

**Fig. 2:**
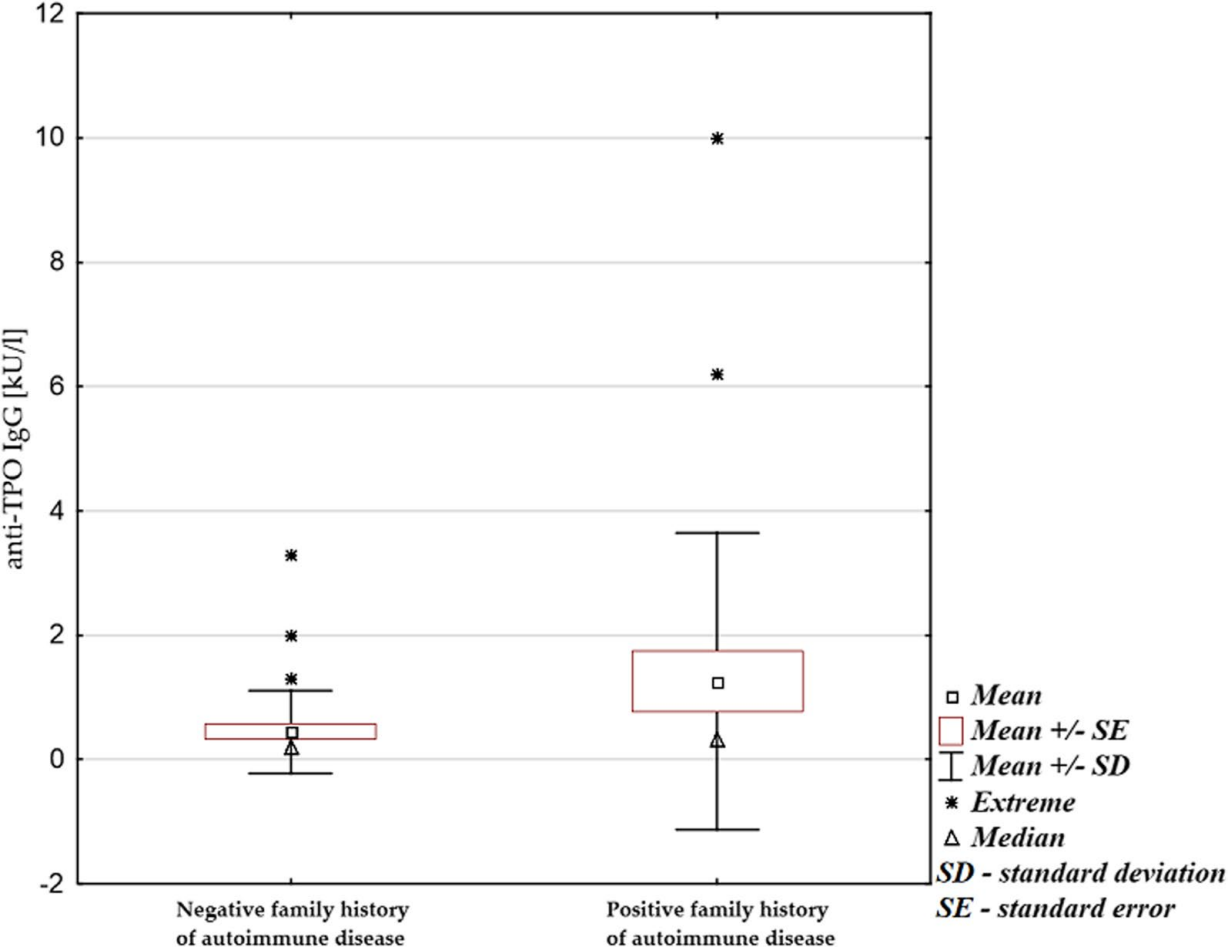
Anti-TPO antibodies level in patients with a positive and negative family history of autoimmune diseases (p = 0.044; Mann–Whitney U test for non-parametric quantitative data)

Eight out of 58 patients (13.8%) had positive (≥ 0.80 kU/L) anti-TPO antibodies, which were the most frequently increased AA. None of these patients were previously diagnosed with Hashimoto’s thyroiditis. Two out of eight patients with positive anti-TPO antibodies were undergoing immunoglobulin replacement therapy (IRT). The difference between patients receiving IRT and those who were not included in the treatment was statistically significant (p = 0.0009; Fig. 1). Anti-TPO antibodies were elevated more often in patients with a positive family history of AD, including Hashimoto’s thyroiditis (n = 3; p = 0.044; Fig. 2). Two out of 10 subjects from the control group had positive anti-TPO, however the level of antibodies was low and clinically insignificant (< 1.00 kU/L).

Positive anti-deamidated gliadin peptide (anti-DGP) antibodies were detected in 6.9% (n = 4) of patients in the study group, two of them had also elevated anti-tissue transglutaminase (anti-tTG) IgG antibodies. Only one of the patients with antibodies specific to coeliac disease (CD) had been previously diagnosed with CD. Although none of the control group had positive anti-tTG and anti-DGP IgA antibodies, the difference between the study and the control group was not statistically significant (p = 0.312). 3.45% of those in the study group (n = 2) had positive anti-Sm antibodies and only one (1.72%) had anti-La/SS-B antibodies.

There was no significant difference between PAD patients and patients with other PIDs with regard to the presence of AA.

There was a significant correlation (p = 0.0009) between positive coeliac antibodies and IgG levels in the study group—patients with positive anti-DGP and/or anti-tTG IgG had normal (n = 3) or increased (n = 1) total IgG levels. A significant difference was observed with regard to IgG1 levels and presence of coeliac autoantibodies (p = 0.00009) as well.

### Clinical manifestations

Among all 58 patients included in the study, only 8.62% (n = 5) had a previous diagnosis of autoimmune disease, including CD (n = 2), autoimmune thrombocytopenia (n = 2) and autoimmune neutropenia (AN; n = 1; Table 2). Most of them were girls (n = 3), but the difference between the sexes was not statistically significant (p = 0.113). Three patients were previously diagnosed with hypothyroidism. Among four patients with positive anti-DGP and/or anti-tTG, only one had been previously diagnosed with CD and IgG subclass deficiency, one was under immunological care due to AN and lymphocyte T deficiency with a history of pyogenic infections, two were complaining of chronic diarrhoea (one of them had selective IgA deficiency and the other IgM deficiency). CD was then confirmed by means of other serological tests (e.g. anti-endomysial antibodies IgA/IgG) and typical histopathological changes were found in duodenal mucosa in two out of three patients.

**Table 2:** Prevalence of autoantibodies and previously diagnosed autoimmune diseases among recruited PID patients

Type of PID (n = 58)	Previous diagnosis of autoimmune disease	Presence of autoantibodies
Combined immunodeficiency with associated or syndromic features (CID; n = 5)	0.00% (n = 0)	20% (n = 1)
Predominantly antibody deficiency* (PAD; n = 46)	8.7% (n = 4)	21.74% (n = 10)
Congenital defects of phagocyte number, function or both (n = 3)	0.00% (n = 0)	33.33% (n = 1)
Complement deficiency (n = 2)	0.00% (n = 0)	0.00% (n = 0)
Others** (n = 2)	50.00% (n = 1)	100.00% (n = 2)

*PAD: CVID/IgG subclass deficiency/IgG subclass deficiency with IgA deficiency/IgG deficiency/IgG and IgA deficiency/IgM deficiency/IgM and IgG subclass deficiency/IgM and IgA deficiency/IgM, IgG and IgA deficiency/selective IgA deficiency/transient hypogammaglobulinemia of infancy/X-linked agammaglobulinemia

**Others: lymphocyte T deficiency/ lymphocyte T deficiency and autoimmune neutropenia

Ab—antibody; CID—combined immunodeficiency; CVID—common variable immunodeficiency; Ig—immunoglobulin; n —number; PAD—predominantly antibody deficiency; PID—primary immunodeficiencies

18.97% (n = 11) of patients with PIDs had hepatomegaly and/or splenomegaly and/or lymphadenopathy (considered as lymphoproliferation below) documented in their medical history. It more often affected boys (n = 10) than girls (n = 1), and the difference between the sexes was statistically significant (p = 0.025). All patients with CVID who took part in the study (n = 3) had developed lymphoproliferation. In two of them, it proved to be the cause of immunological diagnostics (collaterally with recurrent respiratory tract infections).

### Haematological findingss

Among 58 patients in the study group, haematological abnormalities were quite common in the course of the research. Decreased haemoglobin levels were found in 12.07% (n = 7) of patients, leukopenia in 25.86% (n = 15) and neutropenia in 22.41 (n = 13) (antineutrophil antibodies were detected in one case of chronic neutropenia). There were two cases of immune thrombocytopenia. No statistically significant difference was found between patients with positive or negative AA in the presence of leukopenia (p = 0.664), neutropenia (0.526) and anaemia (0.358). Such factors as gender, IRT, recurrent infections, positive family history of autoimmune diseases, lymphoproliferation did not correlate with the presence of anaemia, leukopenia or neutropenia.

## Discussion

The coexistence of immunodeficiency and autoimmunity is an important research field because of the parallel hypoimmune and hyperimmune state one of each other [18, 20]. The main pathophysiologic mechanisms leading to the development of autoimmunity in PIDs are still under debate. Investigation of complex immune regulatory and signalling mechanisms coupled with the genetic analysis, reveals complex relationships between primary immunodeficiency syndromes and autoimmune diseases [12, 21].

It was demonstrated that in PID patients, either tolerance or ignorance may be affected by intense antigen load as a result of recurrent or persistent infections (by molecular mimicry and/or the presence of superantigens) and defects in antigen clearance, which make patients liable to dysregulated immune responses and autoimmune disorders [18]. Defective antigen clearance may result in end-organ deposition of immune complexes, cellular activation, chronic inflammation, and tissue destruction, as well as in the formation of anti-tissue antibodies [18]. In this study, the majority of patients (n = 38) had a history of recurrent infections (i.e. recurrent otitis media/sinusitis/pneumonia/abscesses), yet no statistically significant difference between patients with positive/negative autoantibodies and recurrent infections was found.

Autoimmunity is an easily recognised complication in PID patients, but data about its prevalence among paediatric individuals are limited. Most of the reported studies on the frequency of autoimmune and inflammatory manifestations in PID patients were conducted in adults. A recent French national study by Fischer et al. [14], which is the largest to date and includes all types of PID and autoimmune manifestations, was carried out in both paediatric and adult populations. One or more autoimmune and inflammatory complications were recorded in 26.2% of all subjects with a risk of onset throughout the patient’s lifetime. The study highlighted the increased risk of developing inflammatory bowel disease and arthritis in PID children compared to the general population [14].

In a retrospective study by Kaplan et al. [22] conducted in Turkish children with PID, autoimmune and inflammatory manifestations were observed in 10.1% and the median age of autoimmunity initial time was 61.3 ± 53 months. In comparison to this study, most of the patients were male (55.4%). However, consanguinity was present in the case of 34.9%. The distribution of PID types associated with autoimmunity was different (phagocyte deficiencies—56%, CIDs—53%, immune dysregulation diseases—52%). The most common autoimmune manifestation was autoimmune thyroiditis. Our study group did not include individual diagnosed with documented Hashimoto’s thyroiditis before the study, but three others had hypothyroidism. During the research, anti-TPO antibodies were the most frequently increased ones and statistically occurred more often in children with a positive family history of Hashimoto’s thyroiditis. This group of patients was characterized by a larger risk of potential development of autoimmune thyroiditis and should be monitored in this regard. However, the presence of anti-TPO antibodies in a significant percent of the control group (20%) and the lack of statistical difference between groups challenges anti-TPO as a screening marker in an asymptomatic patients. Presented results should be considered with caution due to the small number of patients. Thereupon, further studies on representative groups are crucial.

In a study by Tahiat et al [23] carried out on Algerian patients diagnosed with PID aged 0.1–80, AA were found in 32.4% of PID patients and 15.8% of healthy controls (p < 0.0005). Anti-nuclear antibodies (ANA) (10.0%) and transglutaminase antibodies (TGA) (8.4%) occurred most frequently. Taking into account that our study group was smaller and consisted of children, ANA were found in 3.45% of patients and TGA in 5.17% (anti-DGP IgG in 6.9%). In Tahiat’s study, almost one-third of patients with positive AA had no autoimmune manifestations, while in this one, 28.57% (4/14) of patients with positive AA were completely asymptomatic in the course of the research. The authors highlighted that positive results regarding AA should be interpreted with caution in patients diagnosed with PIDs due to their low positive predictive value.

It seems paradoxical that AA are produced against self-antigens in patients with CVID, who, at the same time, have low IgG levels and do not produce specific antibodies after vaccinations. In this study, none of the patients with CVID had positive AA. One individual with X-linked agammaglobulinemia had positive anti-TPO antibodies, but this result should be considered with caution since this particular patient was undergoing IRT. Patients with XLA are not expected to produce AA, yet the “leaky” production of autoantibodies and defects in central B-cell tolerance have been reported [24, 25]. The use of intravenous/subcutaneous immunoglobulin in regular therapy may interfere with immunologic tests for AA detection. However, in our study, a generally higher anti-TPO IgG concentration has been recorded in a group that did not undergo IRT.

It has been shown that patients with various PIDs may also develop intestinal inflammation as one of the leading symptoms. Furthermore, it has been demonstrated that individuals diagnosed with CD may have immunodeficiency (i.e. sIgAD; CVID) [18, 26]. Antibodies characteristic of CD were present in 6.9% (n = 4) of patients and only one had been previously diagnosed with this syndrome. The screening for anti-DGP and anti-tTG antibodies in the series identified two earlier undiagnosed CDs. In both cases, CD was confirmed by further testing. Moreover, one patient had sIgAD, a low (1.4 kU/l) level of anti-DGP IgG antibodies and a positive family history of CD, yet further tests did not confirm the diagnosis. Another patient with IgG subclass deficiency had been previously diagnosed with CD and stuck to a gluten-free diet and in this case, antibodies were not detected. Although there was no statistically significant difference between the study group and the control group in regard to coeliac antibodies, it is worth to consider screening for coeliac disease in PID patients. The lack of significant difference between groups probably arised from small number of cases in the control group.

The high prevalence of AD within IgAD and CVID cohorts together with a recent report on the increased prevalence of autoimmunity (10%) in their normoglobulinemic first-degree relatives indicate a role of a common genetic denominator in the induction of these diseases. Studies on the genetic linkage and HLA have demonstrated that IgAD and CVID share a major susceptibility locus in the DQ-DR haplotype on chromosome 6 [27–29]. In our study, 66.67% of patients with sIgAD had positive AA and most of them had a positive family history of AD. Based on the evidence concerning the concurrence of sIgAD and autoimmune disorders, it is worth considering screening patients with sIgAD for autoantibodies.

The IgG subclass deficiency causes susceptibility to infections, however, according to studies, it does not appear to be associated with AD until it co-occurs with IgA deficiency [27, 30, 31]. In our study, 21.05% (n = 4) of patients with isolated IgG subclass deficiency had positive AA and half of them had been previously diagnosed with an AD (coeliac disease in both cases).

The serological diagnosis of most AD is based on AA circulating in the serum and/or plasma and the presence of immune complex deposits containing AA and a complement [18]. Due to the hypogammaglobulinemic condition in individuals diagnosed with PADs and some types of CIDs, diagnostic tests based on antibodies may not be useful in these patients. Specific antibody deficiency (SAD) and CVID are obvious examples of PADs in which the production of AA is low or negative due to dysfunctions of the immune system [32, 33]. Despite a close relationship between the diagnosis of autoimmunity and AA, the results of some PID patients’ tests are persistently negative for disease-specific autoantibodies. On the other hand, multiple studies show that positive AA are more common among PID patients rather than healthy controls [23].

In our study, we found a statistically significant difference between patients with positive and negative coeliac autoantibodies according to IgG level. Higher levels of IgG were observed among PID patients with positive AA. The difference in IgG levels in CVID patients was reported by Boileau et al. [34]—the authors observed a significantly higher IgG levels among CVID patients with autoimmune cytopenia. Other studies have shown that CVID patients with autoimmunity exhibit higher levels of IgM compared to non-autoimmune phenotypes [6, 35]. In this project, the correlation between IgG levels and the presence of autoantibodies should be interpreted in a broad context. Lower IgG levels and a greater percentage of IgG deficiency among patients with negative AA may constitute the cause of negative results of autoantibody tests. In our study, IgM levels did not correlate with the presence of autoantibodies.

Autoimmune diagnostics in patients undergoing IRT seems even more complicated as the use of Ig may interfere with some of the immunologic tests. Therefore, it may be helpful to use frozen serum for future testing if IRT has been initiated [36].

Early onset autoimmunity and autoimmunity that involves multiple organs (known as polyautoimmunity) or significant lymphoproliferation may be signs of an underlying IEI and suggest an immune dysregulation defect. Therefore the awareness of signs of AD and immune dysregulation among patients and clinicians is of the utmost importance. Clinical symptoms, as well as patient personal and family history are crucial in diagnostics and management of patients with IEI. The patients should be closely followed up, especially with regard to their immune status. Combined with genetic information or family history, the presence of elevated levels of autoantibodies may be highly predictive of the later onset of an autoimmune disorder and improve efforts at prevention in individuals at high risk of disease whenever possible. On the other hand, the presence of the AA in a patient does not guarantee a diagnosis of AD and the screening of variable AA might involve considerable cost. Furthermore, autoantibodies can be detected in otherwise healthy individuals and their production is not sufficient for clinical disease. However if the symptoms of an AD occur, especially among patients with already positive AA, an early and proper diagnosis may be helpful to avoid a diagnostic delay. The positive autoantibodies help to support the diagnosis. This is crucial, especially when the treatment requires balancing between increased susceptibility to infections and the additional suppression of the immune system [37].

A main limitation for definitive conclusions was small number of the study and the control group. Since the control group consisted of patients with occasional or mild recurrent infections who did not meet the IUIS criteria for IEI, the comparison between groups should be taken with caution.

## Conclusions

Paediatric PID population requires more detailed assessment of the immune status because autoimmunity often develops at an early age. Selected autoantibodies (i.e. anti-tTG, anti-DGP) may be useful for the screening of PID to avoid a delay of the diagnosis of an autoimmune disease. Early and proper diagnosis can provide treatment options before serious organ damage occurs. Cost-effectiveness analysis should be considered along with the risk factors and clinical symptoms.

## Supplementary Information

**Additional file 1. Table S1. **Control group characteristics.

**Additional file 2. Table S2. **Patients’ baseline characteristics according to PID classification.

## Data Availability

The datasets used and/or analysed during the current study available from the corresponding author on reasonable request. The raw de-identified data may be made available upon reasonable request from the corresponding authors.

## Abbreviations

AA: Autoantibody
AD: Autoimmune disease
AMA-M2: Anti-mitochondrial M2
AN: Autoimmune neutropenia
ANA: Antinuclear antibodies
Anti-Scl-70: Anti-topoisomerase I
A-T: Ataxia–telangiectasia
CD: Coeliac disease
CENP-B: Centromere protein B
CIDs: Combined immunodeficiency disorders
CVID: Common variable immunodeficiency
DGP: Deamidated gliadin peptides
dsDNA: Double-stranded DNA
Hgb: Haemoglobin
HLA: Human leukocyte antigen
ICD-10: International Statistical Classification of Diseases and Related Health Problems
IEI: Inborn errors of immunity
IF: Intrinsic factor
IgE/G/A/M: Immunoglobulin(s) class E/G/A/M
IRT: Immunoglobulin replacement therapy
IUIS: International Union of Immunodeficiency Societies
MPO: Myeloperoxidase
n: Number
PAD: Predominantly antibody deficiencies
PCNA: Proliferating cell nuclear antigen
PIDs: Primary immunodeficiencies
PR3: Proteinase 3
SAD: Specific antibody deficiency
sIgAD: Selective IgA deficiency
SS-A: Sjogren’s syndrome related antigen A
SS-B: Sjogren’s syndrome type B antigen
Sm: Smith antigen
TPO: Thyroid peroxidase
TG: Thyroglobulin
tTG: Tissue transglutaminase
Treg: T regulatory cells
WBC: White blood cell
X-linked: Gene is located in the X chromosome
XLA: X‐linked agammaglobulinemia
