# Supplementary material for: Assessment of autoantibodies in paediatric population with primary immunodeficiencies: a pilot study
Source: BMC Immunol. 2023 Jun 3;24:8. doi: 10.1186/s12865-023-00543-6 (PMC10238767; doi:10.1186/s12865-023-00543-6)
Supplement: Supplementary file 1 — Additional file 1. Table S1. Control group characteristics. [file 12865_2023_543_MOESM1_ESM.docx]

Table S1. Control group characteristics.

| No of patients | No of male | No of female | Median age | Mean age | Immunoglobulin replacement therapy | No of patients with positive autoantibodies | No of patients with positive antibodies for coeliac disease |
| --- | --- | --- | --- | --- | --- | --- | --- |
| n=14 | n=7 (50%) | n=7 (50%) | 7 | 7 | n=0 (0%) | 2/10 (20%) anti-TPO  1F = 0.92 kU/l  2M = 0.96 kU/l | 0/14 (0%) |

Abbreviations: 1F – 1 year old female; 2M – 2 years old male; no-number; TPO - thyroid peroxidase
