# Supplementary material for: Assessment of autoantibodies in paediatric population with primary immunodeficiencies: a pilot study
Source: BMC Immunol. 2023 Jun 3;24:8. doi: 10.1186/s12865-023-00543-6 (PMC10238767; doi:10.1186/s12865-023-00543-6)
Supplement: Supplementary file 2 — Additional file 2. Table S2. Patients’ baseline characteristics according to PID classification. [file 12865_2023_543_MOESM2_ESM.docx]

Table S2. Patients’ baseline characteristics according to PID classification.

| Type of PID | No of patients | No of male | No of female | Median age | Mean age | Immunoglobulin replacement therapy |
| --- | --- | --- | --- | --- | --- | --- |
| Ataxia-telangiectasia | n=3  (5.17% of all) | n=1 (33.33%) | n=2 (66.67%) | 11 | 10 | n=2 (66.67%) |
| DiGeorge syndrome | n=2 (3.45% of all) | n=1 (50.00%) | n=1 (50.00%) | 6 | 6 | n=0 (0%) |
| Predominantly Ab deficiency (n=46):  - CVID (n=3)  - X-linked agammaglobulinemia (n=1)  - other hypo-gammaglobulinemia’s* (n=17)  - IgG subclass deficiency (n=19)  - selective IgA deficiency (n=6) | n=46 (79.31% of all)  n=3 (5.17% of all)  n=1 (1.72% of all)  n= 17 (29.31% of all)  n= 19 (32.76% of all)  n= 6 (10.34% of all) | n= 29 (63.04%)  n=3 (100.00%)  n=1 (100.00%)  n= 12 (68.75%)  n= 9 (47.37%)  n=4 (66.67%) | n= 17 (36.96%)  n=0 (0%)  n=0 (0%)  n= 5 (31.25%)  n= 10 (52.63%)  n=2 (33.33%) | 7  14  15  7  6  10 | 8  13  15  8  6  10 | n=6  n=3 (100.00%)  n=1 (100.00%)  n= 2 (11.76%)  n=0 (0%)  n=0 (0%) |
| Congenital defects of phagocyte number, function or both (n=3) | n= 3 (5.17% of all) | n=2 (66.67%) | n=1 (33.33%) | 4 | 7 | 0% (n=0) |
| Complement deficiency (n=2) | n=2 (3.45% of all) | n=1 (50.00%) | n=1 (50.00%) | 5 | 5 | n=0 (0%) |
| Lymphocyte T deficiency (n=1) | n=1 (1.72% of all) | n=0 (0%) | n=1 (100%) | 10 | 10 | n=0 (0%) |
| Lymphocyte T deficiency and autoimmune neutropenia | n=1 (1.72%) | n=1 (100%) | n=0 (0%) | 3 | 3 | n=0 (0%) |

*Other hypogammaglobulinemia’s: IgG subclass deficiency with IgA deficiency/ IgG deficiency/ IgG and IgA deficiency/ IgM deficiency/ IgM and IgG subclass deficiency/ IgM and IgA deficiency/ IgM, IgG and IgA deficiency/ transient hypogammaglobulinemia of infancy

Abbreviations: Ab – antibody; CVID – common variable immunodeficiency; Ig – immunoglobulin; no-number; PID – primary immunodeficiency
